# 

**Published:** 1893-03

**Authors:** 


Whole Number, CCCLXXVIII.

New Series.

March, 1893.

VOL. XXXII. No. 8.

LIBRARY
Indexed
S. G. O.

### BUFFALO MEDICAL AND SURGICAL JOURNAL.

Established 1845 by AUSTIN FLINT, M. D.

**Editors:**

THOS. LOTHROP, M. D. WM. WARREN POTTER, M. D.

**Associate Editors:**

WILLIAM C. KRAUSS, M. D. JAMES WRIGHT PUTNAM, M. D.

JOHN A. MILLER, Ph. D. DeLANCEY ROCHESTER, M. D.

JOHN PARMENTER, M. D.

SCRIPTA FERUNT ANNOS

European Agency,
TRÜBNER & CO.
57 and 59 Ludgate Hill, London, E. C.

BUFFALO:
1893.

Foreign Subscription, $2.50

Entered at the Post-Office at Buffalo, N. Y., as Second-class Mail Matter.

*Times Printing House, Buffalo, N. Y.*

